# Crystal structure of (20*S*)-21-[4-(2-hy­droxy­propan-2-yl)-1*H*-1,2,3-triazol-4-yl]-20-(4-methyl­pent­yl)-5-pregnen-3β-ol with an unknown solvate

**DOI:** 10.1107/S2056989018003286

**Published:** 2018-03-06

**Authors:** Hugo Santalla, Saray Argibay

**Affiliations:** a Departamento de Química Orgánica, Instituto de Investigación Sanitaria Galicia Sur, Facultade de Química, Universidade de Vigo, E-36310, Vigo, Spain; b Departamento de Química Inorgánica, Instituto de Investigación Sanitaria Galicia Sur, Facultade de Química, Universidade de Vigo, E-36310, Vigo, Spain

**Keywords:** crystal structure, cholesterol, gemini, analogue, hydrogen bonding

## Abstract

In the title cholesterol analogue, [systematic name: (3*S*,8*S*,9*S*,10*R*,13*S*,14*S*,17*R*)-17-{(*S*)-1-[4-(2-hy­droxy­propan-2-yl)-1*H*-1,2,3-triazol-1-yl]-6-methyl­heptan-2-yl}-10,13-dimethyl-2,3,4,7,8,9,10,11,12,13,14,15,16,17-tetra­deca­hydro-1*H*-cyclo­penta­[*a*]phenanthren-3-ol] C_32_H_53_N_3_O_2_, a new chain, including an inter­mediate triazole and a tertiary hydroxyl group in the terminal position, has been added at position 20 inducing a change in its stereochemistry. In the crystal, mol­ecules are linked by O—H⋯O and O—H⋯N hydrogen bonds, forming layers lying parallel to (-201) and enclosing *R* ^4^_4_(36) ring motifs. The isopropyl group is disordered about two positions with a refined occupancy ratio of 0.763 (5):0.237 (5). A region of disordered electron density was corrected for using the SQUEEZE routine in *PLATON* (Spek (2015). *Acta Cryst*. C**71**, 9–18). The given chemical formula and other crystal data do not take into account the unknown solvent mol­ecule(s).

## Abstract 2

In the title analogue of cholesterol, a new chain including an inter­mediate triazole and a tertiary hydroxyl group in the terminal position has been added at position 20, inducing a change in its stereochemistry.

## Chemical context   

The nuclear receptors (NRs) are a large family of ligand-regulated transcriptional factors and include the receptors for steroid hormones, thyroid hormones, lipophilic vitamins and cholesterol metabolites (Mangelsdorf & Evans, 1995 ▸; Burris *et al.*, 2013 ▸). Approximately half of NRs are classified as orphan NRs because they do not have well-characterized ligands (Hummasti & Tontonoz, 2008 ▸). Orphan NRs are an active area of research partly due to their potential for clinical agent development for various diseases (Mohan & Heyman, 2003 ▸). Recent studies have demonstrated that retinoic acid receptor-related orphan receptors (RORs) have been implicated in several physiological and pathological processes.
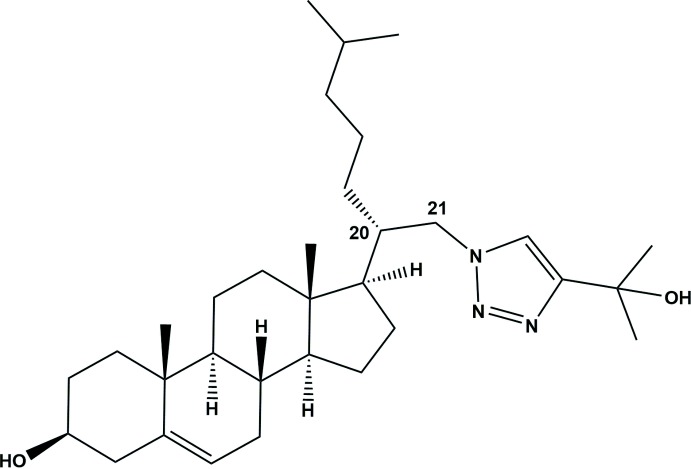


Using the methodology developed in our research group for the synthesis of gemini-type vitamin D analogues (Fall *et al.*, 2011 ▸; Pazos *et al.*, 2016 ▸; Santalla *et al.*, 2017 ▸) (modified with a double side chain), we can access new cholesterol analogues that can be of great inter­est in inter­actions with RORs. In this study, we present the structure of a new analogue of cholesterol (**2**), with eight stereocentres and a double side chain based on the aliphatic chain of cholesterol on the one hand and on the incorporation of a triazole ring on the other, since many aza­steroids have proven to be biologically active. For example, some of them act as 5α-reductase inhibitors, anti­fungal agents and γ-amino­butyric acid (GABA) receptor modulators (Tian *et al.*, 1995 ▸; Burbiel & Bracher, 2003 ▸; Covey *et al.*, 2000 ▸).

## Structural commentary   

**Figure 1:**
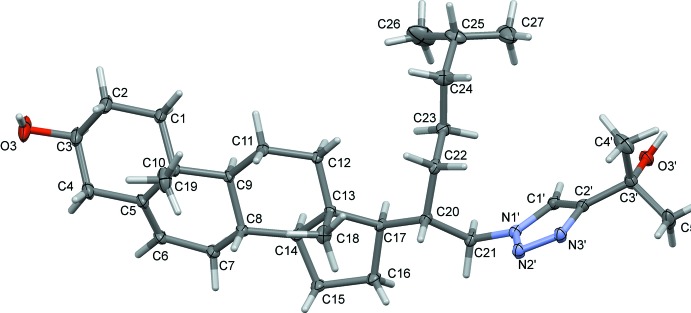
The mol­ecular structure of compound **2**, with the atom labelling. Displacement ellipsoids are drawn at the 30% probability level. In this and other figures the minor disorder component atoms (C24*B*–C27*B*) of the aliphatic chain at C20 have been omitted for clarity.

In the title cholesterol gemini-type analogue **2**, illustrated in Fig. 1 ▸, the four aliphatic rings are structurally identical to those in the cholesterol hormone, *i*-cholesteryl methyl ether (Bernal *et al.*, 1940 ▸; Wang *et al.*, 2014 ▸). In the title compound, atom C20 has a different stereochemistry than in the cholesterol mol­ecule, as a result of stereospecific reactions of the synthetic pathway. Furthermore, a new chain, including an inter­mediate triazole and a tertiary hydroxyl group in the terminal position, has been added at atom C21. Although some steroid analogues with a triazole ring have been synthesized (Seck *et al.*, 2015 ▸), there are no references to any crystallographic analyses of gemini cholesterols with a triazole group at position C21 (Cambridge Structural Database, version 5.39, last update February 2018; Groom *et al.*, 2016 ▸). The terminal OH group (C2′/C3′/O3′) is inclined to the triazole ring (N1′–N3′/C1′/C2′) mean plane by 7.2 (2) °.

## Supra­molecular features   

**Figure 2:**
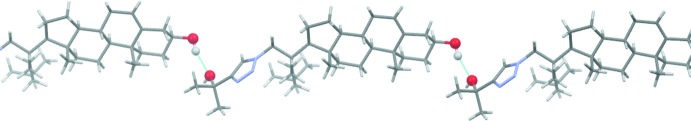
A view of the O—H⋯O hydrogen bonded *C*(18) chain propagating along the [102] direction (blue dashed lines; see Table 1 ▸).

**Figure 3:**
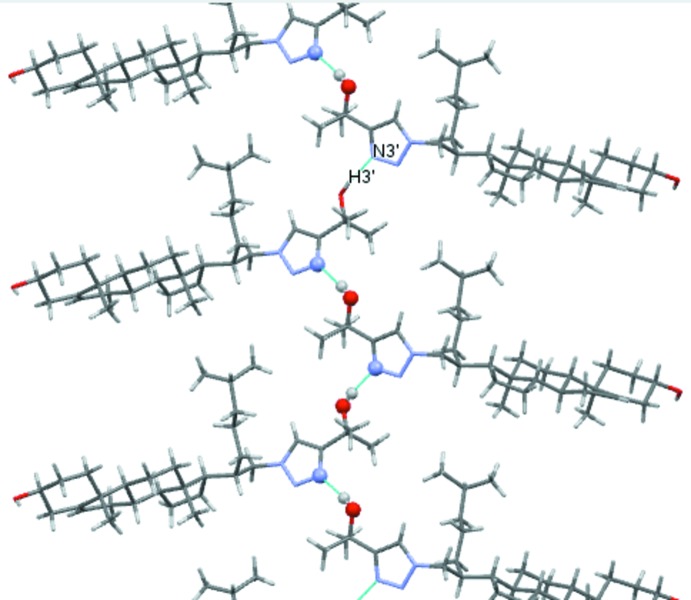
A view of the O—H⋯N hydrogen bonded *C*(5) chain propagating along the [010] direction (blue dashed lines; see Table 1 ▸).

**Figure 4:**
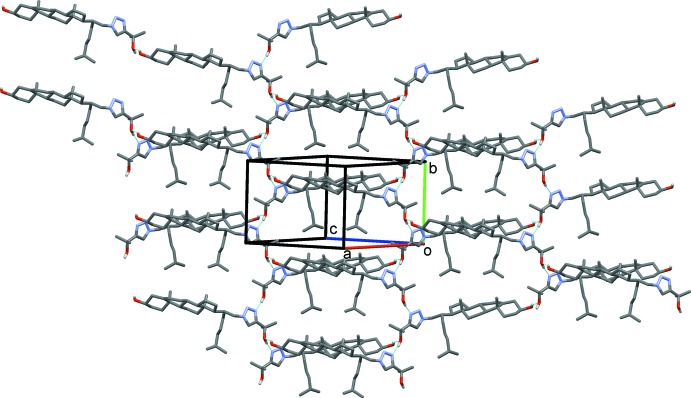
A view approximately normal to the (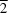
01) plane of the crystal packing of compound **2**. Hydrogen bonds (see Table 1 ▸) are shown as dashed lines, and only H atoms H3 and H3′ have been included.

**Figure 5:**
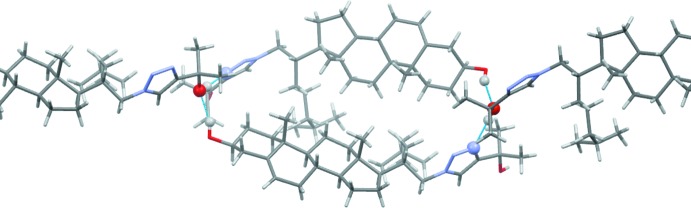
A partial view of the crystal packing of compound **2**, showing details of the O—H⋯O and O—H⋯N hydrogen bonds forming an 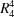
(36) ring motif (blue dashed lines; see Table 1 ▸).

The mol­ecular association in the title compound **2**, is based on hydrogen bonding involving the hydroxyl and triazole groups (Table 1 ▸). These inter­molecular links are present in the form of two chains. The first, a *C*(18) chain (Fig. 2 ▸), is formed by the O3—H3⋯O3^’i^ hydrogen bond with O3—H3 acting as the donor and atom O3′ acting as the acceptor. The second is a *C*(5) chain, in which the triazole group participates, and is formed by hydrogen bond O3′—H3′⋯N3^’ii^ (Fig. 3 ▸); the alcohol group O3′—H3′ acts as the donor towards the acceptor atom N3′. The combination of these inter­actions results in the formation of layers lying parallel to the (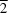
01) plane, as shown in Fig. 4 ▸, and encloses 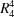
(36) ring motifs, details of which are illustrated in Fig. 5 ▸.

## Synthesis and crystallization   

**Figure 6:**
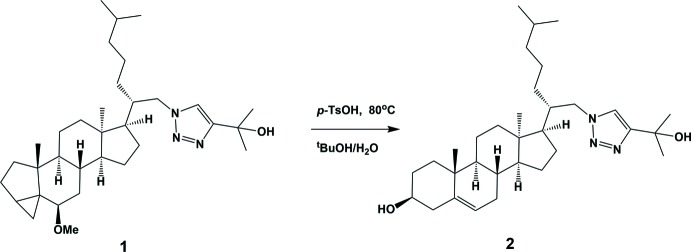
The synthesis of the title compound **2**.

**Compound 2:** details of the synthesis are illustrated in Fig. 6 ▸. To a solution of triazole **1** (12 mg, 0.022 mmol;) in ^*t*^BuOH (2 ml) and water (1 ml) was added *p*-TsOH (5 mg) and the mixture was heated to 353 K for 3 h. The reaction mixture was diluted with water and then extracted with CH_2_Cl_2_ (3 × 5 ml). The combined organic layers were dried with Na_2_SO_4_, filtered, and concentrated. The residue was purified by flash column chromatography (50% EtOAc/hexa­ne) to afford the title diol (11 mg, 99%). Compound **2** was recrystallized as colourless prisms by slow evaporation of a solvent mixture of di­chloro­methane/diethyl ether (1:1) at room temperature [yield 99%; m.p. 778 K; *R*_f_: 0.10 (30% EtOAc/hexa­ne)].

Spectroscopic data for **2**: MS–ESI [*m*/*z* (%)]: 534.40 (10) [*M* ^+^ + Na], 512.42 (100) [*M* ^+^ + H], 494.41 (31) [*M* ^+^ − OMe]. ^1^H NMR (CDCl_3_, δ): 7.36 (1H, *s*, H-1′), 5.35 (1H, *s*, H-6), 4.32 (1H, *m*, H-21), 4.23 (1H, *m*, H-21), 3.52 (1H, *m*, H-3), 2.26 (3H, *m*), 1.94 (5H, *m*), 1.83 (5H, *m*), 1.48 (7H, *m*), 1.27 (4H, *m*), 1.23 (6H, *d*, *J* = 9.2 Hz, CH_3_-4′/5′), 1.06 (3H, *m*), 1.00 (3H, *s*, CH_3_-18), 0.84 (6H, *d*, *J* = 6.6 Hz, CH_3_-26/27), 0.73 (6H, *s*, CH_3_-19) ppm. ^13^C NMR (CDCl_3_, δ): 140.74 (C-5), 128.78 (C-2′), 121.51 (CH-6), 112.41 (C-1′), 77.20 (C-3′), 71.73 (CH-3), 56.38 (CH-14), 52.30 (CH_2_-21), 50.25 (CH), 49.99 (CH), 42.73 (C-13), 42.23 (CH_2_), 41.66 (CH), 39.20 (CH_2_), 39.16 (CH_2_), 37.23 (CH_2_), 36.48 (C-10), 31.93 (CH), 31.80 (CH_2_), 31.61 (CH_2_), 30.50 (CH_3_-4′/5′), 30.47 (CH_3_-4′/5′), 29.30 (CH_2_), 27.85 (CH_2_), 27.82 (CH), 24.26 (CH_2_), 22.69 (CH_3_-26/27), 22.52 (CH_3_-26/27), 22.38 (CH_2_), 21.07 (CH_2_), 19.37 (CH_3_-18), 12.08 (CH_3_-19) p.p.m.

## Refinement   

Crystal data, data collection and structure refinement details are summarized in Table 2 ▸. The O—H and C-bound hydrogen atoms were positioned geometrically (O–H = 0.84 Å, C—H = 0.95–1.00 Å) and refined using a riding model with *U*_iso_(H) = 1.5*U*_eq_(O-hydroxyl, C-meth­yl) and 1.2*U*_eq_(C) for other H atoms. The isopropyl group is disordered about two positions with a refined occupancy ratio of 0.763 (5):0.237 (5) for atoms C24–C27/C24*B*–C27*B*.

A region of disordered electron density was corrected for using the SQUEEZE routine in *PLATON* (Spek, 2015 ▸): volume *ca* 269 Å^3^ for 96 electrons count per unit cell. There is possibly one mol­ecule of diethyl ether per mol­ecule of the title compound **2**. The formula mass and unit-cell characteristics were not taken into account during refinement.

## Supplementary Material

Crystal structure: contains datablock(s) 2, Global. DOI: 10.1107/S2056989018003286/ex2005sup1.cif

CCDC reference: 1825767

Additional supporting information:  crystallographic information; 3D view; checkCIF report

## Figures and Tables

**Table 1:** Hydrogen-bond geometry (Å, °)

*D*—H⋯*A*	*D*—H	H⋯*A*	*D*⋯*A*	*D*—H⋯*A*
O3—H3⋯O3′^i^	0.84	2.00	2.811 (3)	162
O3′—H3′⋯N3′^ii^	0.84	1.97	2.810 (2)	175

Symmetry codes: (i) 
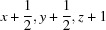
; (ii) 
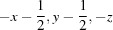
.

**Table 2:** Experimental details

Crystal data	
Chemical formula	C_32_H_53_N_3_O
*M*_r_	511.77
Crystal system, space group	Monoclinic, *C*2
Temperature (K)	100
*a*, *b*, *c* (Å)	20.1130 (15), 10.3898 (7), 15.5934 (12)
β (°)	97.452 (2)
*V* (Å^3^)	3231.0 (4)
*Z*	4
Radiation type	Mo *K*α
μ (mm^−1^)	0.07
Crystal size (mm)	0.35 × 0.30 × 0.24

Data collection	
Diffractometer	Bruker D8 Venture Photon 100 CMOS
Absorption correction	Multi-scan (*SADABS*; Bruker, 2016 ▸)
*T*_min_, *T*_max_	0.688, 0.746
No. of measured, independent and observed [*I* > 2σ(*I*)] reflections	85013, 8040, 7629
*R*_int_	0.029
(sin θ/λ)_max_ (Å^−1^)	0.670

Refinement	
*R*[*F* ^2^ > 2σ(*F* ^2^)], *wR*(*F* ^2^), *S*	0.044, 0.122, 1.02
No. of reflections	8040
No. of parameters	363
No. of restraints	5
H-atom treatment	H-atom parameters constrained
Δρ_max_, Δρ_min_ (e Å^−3^)	0.46, −0.31
Absolute structure	Flack *x* determined using 3430 quotients [(*I* ^+^)−(*I* ^−^)]/[(*I* ^+^)+(*I* ^−^)] (Parsons *et al.*, 2013 ▸)
Absolute structure parameter	0.1 (3)

Computer programs: *APEX3* and *SAINT* (Bruker, 2016 ▸), *SHELXT2014/5* (Sheldrick, 2015*a* ▸), *OLEX2* (Dolomanov *et al.*, 2009 ▸), *Mercury* (Macrae *et al.*, 2008 ▸), *SHELXL2016/6* (Sheldrick, 2015*b* ▸), *PLATON* (Spek, 2009 ▸) and *publCIF* (Westrip, 2010 ▸).

## supplementary crystallographic information

### Crystal data

**Table:**

C_32_H_53_N_3_O	*F*(000) = 1128
*M**_r_* = 511.77	*D*_x_ = 1.052 Mg m^−^^3^
Monoclinic, *C*2	Mo *K*α radiation, λ = 0.71073 Å
*a* = 20.1130 (15) Å	Cell parameters from 9096 reflections
*b* = 10.3898 (7) Å	θ = 2.5–28.4°
*c* = 15.5934 (12) Å	µ = 0.07 mm^−^^1^
β = 97.452 (2)°	*T* = 100 K
*V* = 3231.0 (4) Å^3^	Prism, colourless
*Z* = 4	0.35 × 0.30 × 0.24 mm

### Data collection

**Table:**

Bruker D8 Venture Photon 100 CMOS diffractometer	7629 reflections with *I* > 2σ(*I*)
φ and ω scans	*R*_int_ = 0.029
Absorption correction: multi-scan (SADABS; Bruker, 2016)	θ_max_ = 28.4°, θ_min_ = 2.5°
*T*_min_ = 0.688, *T*_max_ = 0.746	*h* = −26→26
85013 measured reflections	*k* = −13→13
8040 independent reflections	*l* = −20→20

### Refinement

**Table:**

Refinement on *F*^2^	Secondary atom site location: difference Fourier map
Least-squares matrix: full	Hydrogen site location: mixed
*R*[*F*^2^ > 2σ(*F*^2^)] = 0.044	H-atom parameters constrained
*wR*(*F*^2^) = 0.122	*w* = 1/[σ^2^(*F*_o_^2^) + (0.0729*P*)^2^ + 1.6246*P*] where *P* = (*F*_o_^2^ + 2*F*_c_^2^)/3
*S* = 1.02	(Δ/σ)_max_ = 0.008
8040 reflections	Δρ_max_ = 0.46 e Å^−^^3^
363 parameters	Δρ_min_ = −0.31 e Å^−^^3^
5 restraints	Absolute structure: Flack *x* determined using 3430 quotients [(*I*^+^)-(*I*^-^)]/[(*I*^+^)+(*I*^-^)] (Parsons *et al.*, 2013)
Primary atom site location: structure-invariant direct methods	Absolute structure parameter: 0.1 (3)

### Special details

**Table:**

Geometry. All esds (except the esd in the dihedral angle between two l.s. planes) are estimated using the full covariance matrix. The cell esds are taken into account individually in the estimation of esds in distances, angles and torsion angles; correlations between esds in cell parameters are only used when they are defined by crystal symmetry. An approximate (isotropic) treatment of cell esds is used for estimating esds involving l.s. planes.

### Fractional atomic coordinates and isotropic or equivalent isotropic displacement parameters (Å^2^)

**Table:**

	*x*	*y*	*z*	*U*_iso_*/*U*_eq_	Occ. (<1)
O3	0.35759 (11)	0.2665 (4)	0.82807 (13)	0.0775 (9)	
H3	0.329808	0.292008	0.860194	0.116*	
O3'	−0.21535 (8)	−0.17225 (15)	−0.03536 (11)	0.0324 (3)	
H3'	−0.246827	−0.224255	−0.031655	0.049*	
N1'	−0.07599 (8)	0.09363 (17)	0.07066 (10)	0.0246 (3)	
N2'	−0.11583 (9)	0.19700 (17)	0.06509 (12)	0.0296 (4)	
N3'	−0.17644 (9)	0.15741 (18)	0.03258 (12)	0.0291 (4)	
C1	0.21530 (12)	0.1479 (3)	0.66464 (13)	0.0399 (5)	
H1A	0.168814	0.121675	0.670664	0.048*	
H1B	0.238521	0.072198	0.643980	0.048*	
C1'	−0.11028 (11)	−0.0135 (2)	0.04215 (12)	0.0257 (4)	
H1'	−0.093554	−0.098775	0.039622	0.031*	
C2	0.25074 (14)	0.1869 (4)	0.75364 (15)	0.0538 (8)	
H2A	0.226878	0.260248	0.776283	0.065*	
H2B	0.250049	0.114006	0.794424	0.065*	
C2'	−0.17475 (10)	0.02869 (19)	0.01766 (12)	0.0254 (4)	
C3	0.32250 (13)	0.2246 (3)	0.74675 (15)	0.0472 (7)	
H3A	0.346192	0.147179	0.727638	0.057*	
C3'	−0.23710 (11)	−0.0443 (2)	−0.01857 (14)	0.0305 (4)	
C4	0.32511 (12)	0.3296 (3)	0.67895 (15)	0.0388 (5)	
H4A	0.307741	0.410699	0.700939	0.047*	
H4B	0.372452	0.344304	0.670450	0.047*	
C4'	−0.28702 (14)	−0.0460 (3)	0.0473 (2)	0.0491 (7)	
H4'A	−0.328751	−0.087103	0.021575	0.074*	
H4'B	−0.296399	0.042503	0.063909	0.074*	
H4'C	−0.267970	−0.094362	0.098575	0.074*	
C5	0.28505 (10)	0.2978 (2)	0.59230 (13)	0.0290 (4)	
C5'	−0.26936 (15)	0.0171 (3)	−0.1032 (2)	0.0484 (7)	
H5'A	−0.236719	0.019264	−0.144712	0.073*	
H5'B	−0.283572	0.104994	−0.091894	0.073*	
H5'C	−0.308430	−0.033800	−0.127195	0.073*	
C6	0.31207 (10)	0.3053 (2)	0.51844 (14)	0.0313 (4)	
H6	0.358126	0.327892	0.522258	0.038*	
C7	0.27513 (10)	0.2807 (2)	0.43003 (13)	0.0300 (4)	
H7A	0.289590	0.196862	0.408602	0.036*	
H7B	0.286977	0.348379	0.389965	0.036*	
C8	0.19900 (9)	0.27924 (19)	0.42977 (12)	0.0231 (4)	
H8	0.182769	0.369334	0.435870	0.028*	
C9	0.18246 (9)	0.1983 (2)	0.50722 (11)	0.0223 (3)	
H9	0.204785	0.113112	0.502406	0.027*	
C10	0.21297 (10)	0.2561 (2)	0.59538 (13)	0.0275 (4)	
C11	0.10698 (10)	0.1703 (2)	0.50292 (12)	0.0293 (4)	
H11A	0.100051	0.107618	0.548748	0.035*	
H11B	0.083522	0.250810	0.514914	0.035*	
C12	0.07549 (9)	0.1166 (2)	0.41518 (12)	0.0257 (4)	
H12A	0.026664	0.105256	0.415649	0.031*	
H12B	0.095091	0.031154	0.405776	0.031*	
C13	0.08749 (9)	0.20702 (19)	0.34066 (11)	0.0222 (3)	
C14	0.16431 (9)	0.2218 (2)	0.34556 (11)	0.0237 (4)	
H14	0.182393	0.132477	0.342725	0.028*	
C15	0.17510 (11)	0.2859 (2)	0.26021 (13)	0.0320 (4)	
H15A	0.219280	0.262394	0.243283	0.038*	
H15B	0.172312	0.380801	0.264597	0.038*	
C16	0.11729 (10)	0.2325 (2)	0.19454 (13)	0.0300 (4)	
H16A	0.135361	0.177939	0.150946	0.036*	
H16B	0.091499	0.304031	0.164280	0.036*	
C17	0.07153 (8)	0.1516 (2)	0.24737 (11)	0.0212 (3)	
H17	0.088297	0.060868	0.248789	0.025*	
C18	0.05172 (11)	0.3366 (2)	0.34758 (15)	0.0333 (5)	
H18A	0.056778	0.389264	0.296676	0.050*	
H18B	0.071597	0.381738	0.399894	0.050*	
H18C	0.003982	0.321512	0.350577	0.050*	
C19	0.17159 (14)	0.3708 (3)	0.62128 (19)	0.0494 (7)	
H19A	0.164581	0.432269	0.573271	0.074*	
H19B	0.195719	0.413239	0.672214	0.074*	
H19C	0.128087	0.340122	0.634875	0.074*	
C20	−0.00253 (9)	0.1494 (2)	0.20600 (11)	0.0231 (3)	
H20	−0.019403	0.239995	0.205211	0.028*	
C21	−0.00603 (9)	0.1048 (2)	0.11160 (12)	0.0265 (4)	
H21A	0.018056	0.167241	0.078850	0.032*	
H21B	0.016486	0.020320	0.109684	0.032*	
C22	−0.04890 (9)	0.0678 (2)	0.25508 (12)	0.0255 (4)	
H22A	−0.048827	0.105022	0.313526	0.031*	
H22B	−0.095151	0.074933	0.224772	0.031*	
C23	−0.03115 (10)	−0.0746 (2)	0.26492 (14)	0.0304 (4)	
H23A	0.016646	−0.084771	0.288671	0.036*	
H23B	−0.038774	−0.118011	0.207982	0.036*	
C24	−0.0767 (2)	−0.1347 (4)	0.3276 (2)	0.0389 (7)	0.763 (5)
H24A	−0.066712	−0.092988	0.384918	0.047*	0.763 (5)
H24B	−0.124162	−0.116555	0.305481	0.047*	0.763 (5)
C25	−0.06732 (19)	−0.2815 (4)	0.3385 (3)	0.0428 (8)	0.763 (5)
H25	−0.093048	−0.306183	0.386759	0.051*	0.763 (5)
C26	0.0043 (3)	−0.3176 (4)	0.3687 (5)	0.0768 (17)	0.763 (5)
H26A	0.023690	−0.255904	0.412492	0.115*	0.763 (5)
H26B	0.030030	−0.316282	0.319486	0.115*	0.763 (5)
H26C	0.005819	−0.404206	0.393806	0.115*	0.763 (5)
C27	−0.0969 (3)	−0.3579 (8)	0.2627 (4)	0.0667 (14)	0.763 (5)
H27A	−0.069611	−0.347475	0.215460	0.100*	0.763 (5)
H27B	−0.142641	−0.327817	0.243705	0.100*	0.763 (5)
H27C	−0.098069	−0.449028	0.278740	0.100*	0.763 (5)
C24B	−0.0510 (7)	−0.1677 (11)	0.3327 (8)	0.0389 (7)	0.237 (5)
H24B	−0.028169	−0.138464	0.389453	0.047*	0.237 (5)
H24A	−0.099715	−0.156564	0.334308	0.047*	0.237 (5)
C25B	−0.0384 (7)	−0.3100 (11)	0.3271 (8)	0.0428 (8)	0.237 (5)
H25	0.008894	−0.317481	0.314094	0.051*	0.237 (5)
C26B	−0.0377 (6)	−0.3632 (17)	0.4122 (10)	0.073 (5)	0.237 (5)
H26A	−0.079316	−0.340174	0.434835	0.109*	0.237 (5)
H26B	0.000683	−0.328578	0.450387	0.109*	0.237 (5)
H26C	−0.033923	−0.457077	0.409337	0.109*	0.237 (5)
C27B	−0.0815 (13)	−0.359 (3)	0.2463 (14)	0.0667 (14)	0.237 (5)
H27A	−0.098369	−0.285928	0.210137	0.100*	0.237 (5)
H27B	−0.119378	−0.408162	0.262993	0.100*	0.237 (5)
H27C	−0.054504	−0.414775	0.213602	0.100*	0.237 (5)

### Atomic displacement parameters (Å^2^)

**Table:**

	*U*^11^	*U*^22^	*U*^33^	*U*^12^	*U*^13^	*U*^23^
O3	0.0457 (11)	0.155 (3)	0.0296 (9)	−0.0415 (15)	−0.0044 (8)	−0.0143 (13)
O3'	0.0347 (8)	0.0252 (7)	0.0361 (8)	−0.0052 (6)	0.0007 (6)	−0.0033 (6)
N1'	0.0255 (8)	0.0276 (8)	0.0197 (7)	0.0004 (7)	−0.0010 (6)	0.0009 (6)
N2'	0.0305 (9)	0.0242 (8)	0.0308 (8)	0.0007 (7)	−0.0081 (7)	0.0003 (7)
N3'	0.0286 (8)	0.0239 (8)	0.0315 (8)	0.0002 (7)	−0.0087 (7)	0.0007 (7)
C1	0.0368 (11)	0.0615 (15)	0.0200 (9)	−0.0227 (11)	−0.0010 (8)	0.0035 (10)
C1'	0.0320 (10)	0.0240 (9)	0.0206 (8)	0.0007 (7)	0.0009 (7)	−0.0010 (7)
C2	0.0421 (13)	0.097 (2)	0.0215 (10)	−0.0329 (14)	−0.0009 (9)	0.0020 (12)
C2'	0.0296 (10)	0.0249 (9)	0.0205 (8)	−0.0011 (7)	−0.0017 (7)	0.0010 (7)
C3	0.0368 (12)	0.079 (2)	0.0243 (10)	−0.0214 (13)	−0.0031 (9)	−0.0042 (11)
C3'	0.0305 (10)	0.0242 (10)	0.0351 (10)	−0.0036 (8)	−0.0025 (8)	−0.0032 (8)
C4	0.0298 (10)	0.0524 (14)	0.0333 (11)	−0.0169 (10)	0.0006 (8)	−0.0112 (10)
C4'	0.0425 (14)	0.0390 (13)	0.0687 (18)	−0.0098 (11)	0.0181 (13)	−0.0177 (13)
C5	0.0233 (9)	0.0337 (11)	0.0293 (9)	−0.0103 (8)	0.0010 (7)	−0.0031 (8)
C5'	0.0464 (14)	0.0374 (13)	0.0533 (15)	−0.0052 (10)	−0.0250 (12)	0.0007 (11)
C6	0.0219 (9)	0.0403 (12)	0.0314 (10)	−0.0115 (8)	0.0020 (7)	−0.0001 (8)
C7	0.0220 (9)	0.0415 (11)	0.0267 (9)	−0.0094 (8)	0.0042 (7)	0.0023 (8)
C8	0.0203 (8)	0.0254 (9)	0.0237 (8)	−0.0046 (7)	0.0032 (6)	0.0016 (7)
C9	0.0196 (8)	0.0296 (9)	0.0179 (7)	−0.0062 (7)	0.0032 (6)	−0.0027 (7)
C10	0.0235 (9)	0.0367 (11)	0.0223 (8)	−0.0075 (8)	0.0032 (7)	−0.0060 (8)
C11	0.0211 (8)	0.0483 (13)	0.0190 (8)	−0.0097 (8)	0.0048 (6)	−0.0028 (8)
C12	0.0198 (8)	0.0396 (11)	0.0178 (8)	−0.0082 (7)	0.0029 (6)	0.0010 (7)
C13	0.0190 (8)	0.0292 (9)	0.0184 (7)	−0.0006 (7)	0.0026 (6)	0.0003 (7)
C14	0.0195 (8)	0.0331 (10)	0.0190 (8)	−0.0041 (7)	0.0041 (6)	0.0017 (7)
C15	0.0278 (9)	0.0457 (12)	0.0229 (9)	−0.0083 (9)	0.0040 (7)	0.0066 (8)
C16	0.0257 (9)	0.0430 (12)	0.0215 (8)	−0.0027 (8)	0.0042 (7)	0.0063 (8)
C17	0.0181 (8)	0.0287 (9)	0.0171 (7)	0.0013 (7)	0.0032 (6)	0.0018 (7)
C18	0.0301 (10)	0.0339 (11)	0.0349 (10)	0.0067 (9)	0.0007 (8)	−0.0065 (9)
C19	0.0378 (13)	0.0621 (17)	0.0473 (14)	0.0038 (12)	0.0023 (11)	−0.0304 (13)
C20	0.0196 (8)	0.0314 (9)	0.0179 (7)	0.0026 (7)	0.0008 (6)	0.0004 (7)
C21	0.0218 (8)	0.0377 (10)	0.0196 (8)	−0.0001 (8)	0.0015 (6)	−0.0010 (8)
C22	0.0169 (8)	0.0391 (11)	0.0205 (8)	0.0018 (7)	0.0026 (6)	−0.0010 (7)
C23	0.0230 (9)	0.0369 (11)	0.0312 (10)	−0.0016 (8)	0.0031 (7)	0.0063 (8)
C24	0.037 (2)	0.0354 (18)	0.0487 (16)	0.0075 (13)	0.0199 (15)	0.0068 (13)
C25	0.0342 (19)	0.0376 (17)	0.0599 (19)	−0.0024 (14)	0.0192 (15)	0.0051 (14)
C26	0.057 (3)	0.043 (2)	0.129 (5)	0.0094 (19)	0.006 (3)	0.025 (3)
C27	0.080 (4)	0.072 (2)	0.053 (3)	−0.037 (3)	0.029 (2)	−0.010 (2)
C24B	0.037 (2)	0.0354 (18)	0.0487 (16)	0.0075 (13)	0.0199 (15)	0.0068 (13)
C25B	0.0342 (19)	0.0376 (17)	0.0599 (19)	−0.0024 (14)	0.0192 (15)	0.0051 (14)
C26B	0.027 (5)	0.086 (11)	0.103 (12)	0.012 (6)	−0.003 (6)	−0.044 (10)
C27B	0.080 (4)	0.072 (2)	0.053 (3)	−0.037 (3)	0.029 (2)	−0.010 (2)

### Geometric parameters (Å, º)

**Table:**

O3—C3	1.437 (3)	C13—C17	1.559 (2)
O3—H3	0.8400	C14—C15	1.529 (3)
O3'—C3'	1.434 (3)	C14—H14	1.0000
O3'—H3'	0.8400	C15—C16	1.549 (3)
N1'—N2'	1.336 (2)	C15—H15A	0.9900
N1'—C1'	1.354 (3)	C15—H15B	0.9900
N1'—C21	1.472 (2)	C16—C17	1.559 (3)
N2'—N3'	1.324 (2)	C16—H16A	0.9900
N3'—C2'	1.359 (3)	C16—H16B	0.9900
C1—C2	1.530 (3)	C17—C20	1.545 (2)
C1—C10	1.555 (3)	C17—H17	1.0000
C1—H1A	0.9900	C18—H18A	0.9800
C1—H1B	0.9900	C18—H18B	0.9800
C1'—C2'	1.375 (3)	C18—H18C	0.9800
C1'—H1'	0.9500	C19—H19A	0.9800
C2—C3	1.513 (3)	C19—H19B	0.9800
C2—H2A	0.9900	C19—H19C	0.9800
C2—H2B	0.9900	C20—C21	1.536 (3)
C2'—C3'	1.511 (3)	C20—C22	1.536 (3)
C3—C4	1.525 (4)	C20—H20	1.0000
C3—H3A	1.0000	C21—H21A	0.9900
C3'—C4'	1.526 (4)	C21—H21B	0.9900
C3'—C5'	1.532 (3)	C22—C23	1.525 (3)
C4—C5	1.517 (3)	C22—H22A	0.9900
C4—H4A	0.9900	C22—H22B	0.9900
C4—H4B	0.9900	C23—C24B	1.524 (11)
C4'—H4'A	0.9800	C23—C24	1.554 (4)
C4'—H4'B	0.9800	C23—H23A	0.9901
C4'—H4'C	0.9800	C23—H23B	0.9899
C5—C6	1.338 (3)	C24—C25	1.544 (5)
C5—C10	1.520 (3)	C24—H24A	0.9900
C5'—H5'A	0.9800	C24—H24B	0.9900
C5'—H5'B	0.9800	C25—C27	1.484 (7)
C5'—H5'C	0.9800	C25—C26	1.504 (6)
C6—C7	1.501 (3)	C25—H25	1.0000
C6—H6	0.9500	C26—H26A	0.9800
C7—C8	1.531 (3)	C26—H26B	0.9800
C7—H7A	0.9900	C26—H26C	0.9800
C7—H7B	0.9900	C27—H27A	0.9800
C8—C14	1.526 (3)	C27—H27B	0.9800
C8—C9	1.543 (3)	C27—H27C	0.9800
C8—H8	1.0000	C24B—C25B	1.504 (15)
C9—C11	1.539 (3)	C24B—H24B	0.9900
C9—C10	1.552 (2)	C24B—H24A	0.9900
C9—H9	1.0000	C25B—C26B	1.436 (19)
C10—C19	1.538 (3)	C25B—C27B	1.52 (2)
C11—C12	1.536 (3)	C25B—H25	1.0000
C11—H11A	0.9900	C26B—H26A	0.9800
C11—H11B	0.9900	C26B—H26B	0.9800
C12—C13	1.537 (3)	C26B—H26C	0.9800
C12—H12A	0.9900	C27B—H27A	0.9800
C12—H12B	0.9900	C27B—H27B	0.9800
C13—C18	1.537 (3)	C27B—H27C	0.9800
C13—C14	1.545 (2)		
			
C3—O3—H3	109.5	C15—C14—H14	105.8
C3'—O3'—H3'	109.5	C13—C14—H14	105.8
N2'—N1'—C1'	111.34 (16)	C14—C15—C16	103.90 (16)
N2'—N1'—C21	119.75 (17)	C14—C15—H15A	111.0
C1'—N1'—C21	128.66 (18)	C16—C15—H15A	111.0
N3'—N2'—N1'	106.85 (16)	C14—C15—H15B	111.0
N2'—N3'—C2'	109.22 (17)	C16—C15—H15B	111.0
C2—C1—C10	114.0 (2)	H15A—C15—H15B	109.0
C2—C1—H1A	108.7	C15—C16—C17	106.74 (15)
C10—C1—H1A	108.7	C15—C16—H16A	110.4
C2—C1—H1B	108.7	C17—C16—H16A	110.4
C10—C1—H1B	108.7	C15—C16—H16B	110.4
H1A—C1—H1B	107.6	C17—C16—H16B	110.4
N1'—C1'—C2'	104.51 (18)	H16A—C16—H16B	108.6
N1'—C1'—H1'	127.7	C20—C17—C16	112.98 (15)
C2'—C1'—H1'	127.7	C20—C17—C13	117.75 (14)
C3—C2—C1	109.9 (2)	C16—C17—C13	103.26 (15)
C3—C2—H2A	109.7	C20—C17—H17	107.4
C1—C2—H2A	109.7	C16—C17—H17	107.4
C3—C2—H2B	109.7	C13—C17—H17	107.4
C1—C2—H2B	109.7	C13—C18—H18A	109.5
H2A—C2—H2B	108.2	C13—C18—H18B	109.5
N3'—C2'—C1'	108.07 (18)	H18A—C18—H18B	109.5
N3'—C2'—C3'	121.33 (19)	C13—C18—H18C	109.5
C1'—C2'—C3'	130.60 (19)	H18A—C18—H18C	109.5
O3—C3—C2	112.3 (2)	H18B—C18—H18C	109.5
O3—C3—C4	109.8 (2)	C10—C19—H19A	109.5
C2—C3—C4	110.7 (2)	C10—C19—H19B	109.5
O3—C3—H3A	108.0	H19A—C19—H19B	109.5
C2—C3—H3A	108.0	C10—C19—H19C	109.5
C4—C3—H3A	108.0	H19A—C19—H19C	109.5
O3'—C3'—C2'	105.98 (17)	H19B—C19—H19C	109.5
O3'—C3'—C4'	110.79 (19)	C21—C20—C22	110.83 (17)
C2'—C3'—C4'	109.92 (19)	C21—C20—C17	109.05 (14)
O3'—C3'—C5'	109.48 (19)	C22—C20—C17	114.50 (15)
C2'—C3'—C5'	110.59 (19)	C21—C20—H20	107.4
C4'—C3'—C5'	110.0 (2)	C22—C20—H20	107.4
C5—C4—C3	113.54 (19)	C17—C20—H20	107.4
C5—C4—H4A	108.9	N1'—C21—C20	111.20 (15)
C3—C4—H4A	108.9	N1'—C21—H21A	109.4
C5—C4—H4B	108.9	C20—C21—H21A	109.4
C3—C4—H4B	108.9	N1'—C21—H21B	109.4
H4A—C4—H4B	107.7	C20—C21—H21B	109.4
C3'—C4'—H4'A	109.5	H21A—C21—H21B	108.0
C3'—C4'—H4'B	109.5	C23—C22—C20	115.88 (16)
H4'A—C4'—H4'B	109.5	C23—C22—H22A	108.3
C3'—C4'—H4'C	109.5	C20—C22—H22A	108.3
H4'A—C4'—H4'C	109.5	C23—C22—H22B	108.3
H4'B—C4'—H4'C	109.5	C20—C22—H22B	108.3
C6—C5—C4	121.72 (18)	H22A—C22—H22B	107.4
C6—C5—C10	122.64 (18)	C24B—C23—C22	127.3 (5)
C4—C5—C10	115.64 (18)	C22—C23—C24	107.7 (2)
C3'—C5'—H5'A	109.5	C24B—C23—H23A	90.1
C3'—C5'—H5'B	109.5	C22—C23—H23A	110.2
H5'A—C5'—H5'B	109.5	C24—C23—H23A	110.2
C3'—C5'—H5'C	109.5	C24B—C23—H23B	108.0
H5'A—C5'—H5'C	109.5	C22—C23—H23B	110.2
H5'B—C5'—H5'C	109.5	C24—C23—H23B	110.2
C5—C6—C7	124.95 (18)	H23A—C23—H23B	108.5
C5—C6—H6	117.5	C25—C24—C23	113.1 (3)
C7—C6—H6	117.5	C25—C24—H24A	109.0
C6—C7—C8	112.33 (17)	C23—C24—H24A	109.0
C6—C7—H7A	109.1	C25—C24—H24B	109.0
C8—C7—H7A	109.1	C23—C24—H24B	109.0
C6—C7—H7B	109.1	H24A—C24—H24B	107.8
C8—C7—H7B	109.1	C27—C25—C26	112.6 (5)
H7A—C7—H7B	107.9	C27—C25—C24	114.2 (5)
C14—C8—C7	110.59 (16)	C26—C25—C24	112.3 (3)
C14—C8—C9	109.85 (15)	C27—C25—H25	105.6
C7—C8—C9	108.69 (16)	C26—C25—H25	105.6
C14—C8—H8	109.2	C24—C25—H25	105.6
C7—C8—H8	109.2	C25—C26—H26A	109.5
C9—C8—H8	109.2	C25—C26—H26B	109.5
C11—C9—C8	112.38 (15)	H26A—C26—H26B	109.5
C11—C9—C10	112.73 (15)	C25—C26—H26C	109.5
C8—C9—C10	112.47 (15)	H26A—C26—H26C	109.5
C11—C9—H9	106.2	H26B—C26—H26C	109.5
C8—C9—H9	106.2	C25—C27—H27A	109.5
C10—C9—H9	106.2	C25—C27—H27B	109.5
C5—C10—C19	109.78 (19)	H27A—C27—H27B	109.5
C5—C10—C9	110.59 (15)	C25—C27—H27C	109.5
C19—C10—C9	111.55 (18)	H27A—C27—H27C	109.5
C5—C10—C1	106.57 (17)	H27B—C27—H27C	109.5
C19—C10—C1	110.3 (2)	C25B—C24B—C23	121.4 (9)
C9—C10—C1	107.97 (17)	C25B—C24B—H24B	107.0
C12—C11—C9	113.46 (15)	C23—C24B—H24B	107.0
C12—C11—H11A	108.9	C25B—C24B—H24A	107.0
C9—C11—H11A	108.9	C23—C24B—H24A	107.0
C12—C11—H11B	108.9	H24B—C24B—H24A	106.7
C9—C11—H11B	108.9	C26B—C25B—C24B	107.8 (12)
H11A—C11—H11B	107.7	C26B—C25B—C27B	124.9 (14)
C11—C12—C13	111.36 (16)	C24B—C25B—C27B	107.2 (17)
C11—C12—H12A	109.4	C26B—C25B—H25	105.1
C13—C12—H12A	109.4	C24B—C25B—H25	105.1
C11—C12—H12B	109.4	C27B—C25B—H25	105.1
C13—C12—H12B	109.4	C25B—C26B—H26A	109.5
H12A—C12—H12B	108.0	C25B—C26B—H26B	109.5
C18—C13—C12	111.19 (16)	H26A—C26B—H26B	109.5
C18—C13—C14	112.56 (17)	C25B—C26B—H26C	109.5
C12—C13—C14	106.11 (14)	H26A—C26B—H26C	109.5
C18—C13—C17	110.35 (16)	H26B—C26B—H26C	109.5
C12—C13—C17	116.48 (16)	C25B—C27B—H27A	109.5
C14—C13—C17	99.58 (14)	C25B—C27B—H27B	109.5
C8—C14—C15	118.56 (17)	H27A—C27B—H27B	109.5
C8—C14—C13	115.10 (15)	C25B—C27B—H27C	109.5
C15—C14—C13	104.73 (15)	H27A—C27B—H27C	109.5
C8—C14—H14	105.8	H27B—C27B—H27C	109.5
			
C1'—N1'—N2'—N3'	−0.2 (2)	C8—C9—C11—C12	50.0 (2)
C21—N1'—N2'—N3'	−174.88 (16)	C10—C9—C11—C12	178.37 (18)
N1'—N2'—N3'—C2'	0.0 (2)	C9—C11—C12—C13	−55.9 (2)
N2'—N1'—C1'—C2'	0.2 (2)	C11—C12—C13—C18	−64.8 (2)
C21—N1'—C1'—C2'	174.35 (17)	C11—C12—C13—C14	57.9 (2)
C10—C1—C2—C3	−59.8 (4)	C11—C12—C13—C17	167.62 (16)
N2'—N3'—C2'—C1'	0.1 (2)	C7—C8—C14—C15	−58.9 (2)
N2'—N3'—C2'—C3'	179.24 (18)	C9—C8—C14—C15	−178.86 (17)
N1'—C1'—C2'—N3'	−0.2 (2)	C7—C8—C14—C13	176.05 (17)
N1'—C1'—C2'—C3'	−179.2 (2)	C9—C8—C14—C13	56.1 (2)
C1—C2—C3—O3	177.9 (3)	C18—C13—C14—C8	61.5 (2)
C1—C2—C3—C4	54.9 (4)	C12—C13—C14—C8	−60.4 (2)
N3'—C2'—C3'—O3'	173.26 (18)	C17—C13—C14—C8	178.33 (16)
C1'—C2'—C3'—O3'	−7.8 (3)	C18—C13—C14—C15	−70.5 (2)
N3'—C2'—C3'—C4'	−67.0 (3)	C12—C13—C14—C15	167.64 (17)
C1'—C2'—C3'—C4'	111.9 (3)	C17—C13—C14—C15	46.35 (19)
N3'—C2'—C3'—C5'	54.7 (3)	C8—C14—C15—C16	−162.71 (18)
C1'—C2'—C3'—C5'	−126.4 (3)	C13—C14—C15—C16	−32.7 (2)
O3—C3—C4—C5	−175.9 (2)	C14—C15—C16—C17	5.9 (2)
C2—C3—C4—C5	−51.3 (3)	C15—C16—C17—C20	150.76 (18)
C3—C4—C5—C6	−128.2 (3)	C15—C16—C17—C13	22.5 (2)
C3—C4—C5—C10	51.2 (3)	C18—C13—C17—C20	−48.0 (2)
C4—C5—C6—C7	−177.4 (2)	C12—C13—C17—C20	80.0 (2)
C10—C5—C6—C7	3.2 (4)	C14—C13—C17—C20	−166.57 (17)
C5—C6—C7—C8	14.6 (3)	C18—C13—C17—C16	77.23 (19)
C6—C7—C8—C14	−165.92 (18)	C12—C13—C17—C16	−154.78 (16)
C6—C7—C8—C9	−45.3 (2)	C14—C13—C17—C16	−41.30 (18)
C14—C8—C9—C11	−48.2 (2)	C16—C17—C20—C21	53.8 (2)
C7—C8—C9—C11	−169.37 (17)	C13—C17—C20—C21	174.09 (17)
C14—C8—C9—C10	−176.74 (16)	C16—C17—C20—C22	178.58 (17)
C7—C8—C9—C10	62.1 (2)	C13—C17—C20—C22	−61.1 (2)
C6—C5—C10—C19	−111.7 (3)	N2'—N1'—C21—C20	59.8 (2)
C4—C5—C10—C19	68.8 (3)	C1'—N1'—C21—C20	−113.9 (2)
C6—C5—C10—C9	11.8 (3)	C22—C20—C21—N1'	49.7 (2)
C4—C5—C10—C9	−167.7 (2)	C17—C20—C21—N1'	176.60 (17)
C6—C5—C10—C1	128.9 (2)	C21—C20—C22—C23	63.9 (2)
C4—C5—C10—C1	−50.6 (3)	C17—C20—C22—C23	−60.0 (2)
C11—C9—C10—C5	−172.73 (18)	C20—C22—C23—C24B	157.7 (7)
C8—C9—C10—C5	−44.4 (2)	C20—C22—C23—C24	171.5 (2)
C11—C9—C10—C19	−50.3 (3)	C22—C23—C24—C25	176.0 (3)
C8—C9—C10—C19	78.0 (2)	C23—C24—C25—C27	−73.4 (4)
C11—C9—C10—C1	71.0 (2)	C23—C24—C25—C26	56.4 (5)
C8—C9—C10—C1	−160.65 (18)	C22—C23—C24B—C25B	171.1 (8)
C2—C1—C10—C5	55.4 (3)	C23—C24B—C25B—C26B	158.2 (11)
C2—C1—C10—C19	−63.7 (3)	C23—C24B—C25B—C27B	−65.1 (15)
C2—C1—C10—C9	174.2 (2)		

### Hydrogen-bond geometry (Å, º)

**Table:**

*D*—H···*A*	*D*—H	H···*A*	*D*···*A*	*D*—H···*A*
O3—H3···O3′^i^	0.84	2.00	2.811 (3)	162
O3′—H3′···N3′^ii^	0.84	1.97	2.810 (2)	175

Symmetry codes: (i) *x*+1/2, *y*+1/2, *z*+1; (ii) −*x*−1/2, *y*−1/2, −*z*.

## References

[bb1] Bernal, J. D., Crowfoot, D. & Fankuchen, I. (1940). *Philos. Trans. Roy. Soc. A: Math. Phys. Engineering Sci.* **239**, 135–182.

[bb2] Bruker (2016). *APEX3*, *SAINT* and *SADABS*. Bruker AXS Inc., Madison, Wisconsin, USA.

[bb3] Burbiel, J. & Bracher, F. (2003). *Steroids*, **68**, 587–594. 10.1016/s0039-128x(03)00080-1 12957663

[bb4] Burris, T. P., Solt, L. A., Wang, Y., Crumbley, C., Banerjee, S., Griffett, K., Lundasen, T., Hughes, T. & Kojetin, D. J. (2013). *Pharmacol. Rev.* **65**, 710–778. 10.1124/pr.112.006833 PMC11060414 23457206

[bb5] Covey, D. F., Han, M., Kumar, A. S., de la Cruz, M. A. M., Meadows, E. S., Hu, Y., Tonnies, A., Nathan, D., Coleman, M., Benz, A., Evers, A. S., Zorumski, C. F. & Mennerick, S. (2000). *J. Med. Chem.* **43**, 3201–3204. 10.1021/jm0002477 10966737

[bb6] Dolomanov, O. V., Bourhis, L. J., Gildea, R. J., Howard, J. A. K. & Puschmann, H. (2009). *J. Appl. Cryst.* **42**, 339–341.

[bb7] Fall, Y., Gómez, G., Pérez, M., Gándara, Z., Pérez, X., Pazos, G. & Kurz, G. (2011). PCT Int. Appl. WO2011121152A120111006.

[bb8] Groom, C. R., Bruno, I. J., Lightfoot, M. P. & Ward, S. C. (2016). *Acta Cryst.* B**72**, 171–179. 10.1107/S2052520616003954 PMC4822653 27048719

[bb9] Hummasti, S. & Tontonoz, P. (2008). *Mol. Endocrinol.* **22**, 1743–1753. 10.1210/me.2007-0566 PMC2505328 18258684

[bb10] Macrae, C. F., Bruno, I. J., Chisholm, J. A., Edgington, P. R., McCabe, P., Pidcock, E., Rodriguez-Monge, L., Taylor, R., van de Streek, J. & Wood, P. A. (2008). *J. Appl. Cryst.* **41**, 466–470.

[bb11] Mangelsdorf, D. J. & Evans, R. M. (1995). *Cell*, **83**, 841–850. 10.1016/0092-8674(95)90200-7 8521508

[bb12] Mohan, R. & Heyman, R. A. (2003). *Curr. Top. Med. Chem.* **3**, 1637–1647. 10.2174/1568026033451709 14683519

[bb13] Parsons, S., Flack, H. D. & Wagner, T. (2013). *Acta Cryst.* B**69**, 249–259. 10.1107/S2052519213010014 PMC3661305 23719469

[bb14] Pazos, G., Pérez, M., Gándara, Z., Gómez, G. & Fall, Y. (2016). *RSC Adv.* **6**, 61073–61076.

[bb15] Santalla, H., Martínez, A., Garrido, F., Gómez, G. & Fall, Y. (2017). *Org. Chem. Front.* **4**, 1999–2001.

[bb16] Seck, I., Fall, A., Lago, C., Sène, M., Gaye, M., Seck, M., Gómez, G. & Fall, Y. (2015). *Synthesis*, **47**, 2826–2830.

[bb17] Sheldrick, G. M. (2015*a*). *Acta Cryst.* A**71**, 3–8.

[bb18] Sheldrick, G. M. (2015*b*). *Acta Cryst.* C**71**, 3–8.

[bb19] Spek, A. L. (2009). *Acta Cryst.* D**65**, 148–155. 10.1107/S090744490804362X PMC2631630 19171970

[bb20] Spek, A. L. (2015). *Acta Cryst.* C**71**, 9–18. 10.1107/S2053229614024929 25567569

[bb21] Tian, G., Mook, R., Moss, M. L. & Frye, S. V. (1995). *Biochemistry*, **34**, 13453–13459. 10.1021/bi00041a024 7577933

[bb22] Wang, J.-R., Zhou, C., Yu, X. & Mei, X. (2014). *Chem. Commun.* **50**, 855–858. 10.1039/c3cc47747a 24296723

[bb23] Westrip, S. P. (2010). *J. Appl. Cryst.* **43**, 920–925.

